# Exploring paramedic perspectives on emergency medical service (EMS) delivery in Alberta: a qualitative study

**DOI:** 10.1186/s12873-024-00986-z

**Published:** 2024-04-16

**Authors:** Janna Newton, Travis Carpenter, Jennifer Zwicker

**Affiliations:** 1 University of Calgary, Calgary, AB Canada; 2 University of Toronto, Toronto, ON Canada

**Keywords:** Pre-hospital, Emergency medical services, Paramedics, Emergency medical dispatch, Health policy, Scope of practice, Capacity building

## Abstract

**Purpose:**

Emergency Medical Services (EMS) in Alberta are facing critical challenges. This qualitative study aims to describe and understand the frontline perspective regarding system level issues and propose provider-informed policy recommendations.

**Methods:**

19 semi-structured one-on- one interviews were conducted with Primary or Advanced Care Paramedics (PCP/ACP) across Alberta. Participants were asked to share their perspectives, experiences and recommendations in relation to EMS response times and the working environment. Interviews were analyzed using thematic analysis to identify themes and subthemes.

**Results:**

Two core themes were identified as areas of concern: poor response times and the EMS working environment, which each influence and impact the other. Within response times, paramedics highlighted specific difficulties with ED offloading, a lack of resources, low-acuity calls, and rural challenges. In terms of the EMS working environment, four subthemes were apparent including attrition, unhealthy culture, organizational barriers and the need for paramedic empowerment. Providers made many recommendations including creating and expanding emergency mobile integrated health (MIH) branches, sharing 811 and 911 responses, and enforcing ED target offload times amongst other suggestions.

**Conclusions:**

While response times are a key and highly visible problem, there are many critical factors like the EMS working environment that degrade patient care and cause concern amongst frontline practitioners. Multifaceted policy changes are to be explored to reduce disfunction within EMS services, enhance the well-being of the workforce and deliver improved patient care. Specific EMS-oriented policies are important for moving forward to reduce transfers to EDs, but the broader health system which is over capacity is causing downstream effects into EMS must be addressed by government and health administrators.

**Supplementary Information:**

The online version contains supplementary material available at 10.1186/s12873-024-00986-z.

## Introduction

EMS response times are crucial in life-threatening emergencies [1]. Poor response times reflect specific difficulties with operational processes such as prolonged out-of-service times for ambulances, inadequate staffing levels, or inefficient basing and dispatching [2, 3]. In a 2019 Alberta study, 65% of high-acuity patients who arrived at a hospital experienced delays of 30 or more minutes, highlighting chronic concerns about inadequate response times [4]. Alberta has a history of attempting to effectively manage this issue, in 2009 taking an approach of implementing a borderless EMS system aimed at significantly enhancing service delivery by optimizing resource availability and use across the province. This also formally integrated paramedics into the healthcare system. In the context of the anticipated benefits of this policy generally not materializing as expected, decreasing response times remains a government priority. Academically, it is important to analyze why response times remain poor and any unintended consequences seen with these policy changes, particularly from those who know the system functionality best. Since EMS is an understudied field, the basis of evidence needs to be developed and a frontline perspective incorporated to understand the optimal role of paramedics in the healthcare system.

Beyond the importance of improving response times for patient care, it is also important to reduce the out-of-service time for ambulances and increase the number of available resources to, in part, facilitate improved response times [5, 6]. Alberta continues to experience acute shortages in the labour that is the foundation of the EMS workforce. Each week approximately 300–400 paramedic shifts are unfilled, resulting in current staff taking on considerable overtime to compensate [7]. In 2022 overall, 22,000 paramedic shifts went unfilled due to sick time and burnout [8]. Some sources highlight a concerning potential trend where rural ambulances are called into short-staffed cities, potentially leaving rural communities without EMS coverage for hours, which also can push crews into overtime [9, 10]. In this setting, ensuring adequate recruitment and retention of paramedic practitioners is crucial to maintaining a functional EMS system. In 2022, a 10-point plan was established by Alberta Health Services (AHS) to deal with the increased and sustained emergency medical call volume, poor response times and overall lack of resources. Through multiple different policies, the plan aimed to begin reducing system pressure and building capacity by redirecting low acuity calls, implementing integrated operation centres in Calgary and Edmonton, diverting preassigned ambulances to higher acuity calls, and a metro response plan to address rural ambulances in urban centres. This paper is focused on understanding provider perspectives on how to continue addressing the worsening EMS crisis in Alberta [11]. At this pivotal moment, incorporating and understanding the perspectives of paramedics on the frontline is needed to adequately characterize operational concerns and identify factors likely to influence the rollout of successful policies to address this crisis.

This qualitative study aimed to identify and build a deeper understanding of core issues impacting EMS services in Alberta, using insight from frontline paramedic providers to accurately describe the current state of the Alberta EMS system which is typically an underresearched area. Proposals were sought for policy solutions likely to be effective and sustainable in improving EMS service delivery and health system function overall.

## Methods

### Study design

This study used a qualitative research design and interviews to better understand the experiences and perspectives of paramedics in Alberta. The study used an interpretive description methodology to analyze the data to help gain insights into individuals’ perspectives and experiences [12]. This study received approval from the University of Calgary Conjoint Health Research Ethics Board (CHREB, REB22-1703) and informed consent to participate was obtained before all interviews.

### Participants

Paramedics were recruited via purposeful, maximum variation sampling. Based on the defined study population, recruitment was targeted to obtain a representative sample of paramedics with the desired years of service, professional designation, and practice setting. A recruitment poster was posted on multiple social media platforms with the eligibility criteria and contact information for those interested in participating. The selection of subjects by the researchers was tailored to ensure varied demographics including inclusion criteria focused on individuals who were registered as a Primary or Advanced Care Paramedic (PCP/ACP) in Alberta, had a minimum of 2000 h working as paramedics and had provided frontline service in the last five years (Jan 1, 2018 – Jan 1, 2023). Participants were continually recruited until new data did not significantly add to existing themes, thereby identifying core themes [13].

### Data collection

Participants were all interviewed by the corresponding author over Zoom™ using the interview guide (Table 1) which was developed in collaboration with prehospital and research experts from a multidisciplinary team. Questions were designed to be semi-structured and open for the participant to express their experience, probes were used to give examples or a starting point to discuss their experience as a paramedic. Follow-up or probing questions were added for clarification. The interview guide was tested with a research associate, and subsequent modifications were made to improve the clarity of questions. The target time of each interview was between 60 and 90 min. The one-on-one interview focused on three themes: response time, ED offloading, and EMS working environment (Table 1, Appendix A-B).

**Table 1:** Interview questions

Theme	Questions	Probes
Warm-Up	1. Can you tell me what your designation is? OR You indicated that you are a PCP/ACP, can you confirm that this is correct? 2. Can you please tell me about your role(s) with EMS? 3. In your time working for EMS, what has the culture been like? Note: Culture here is defined as a collection of attitudes, beliefs, and behaviours that make up the workplace environment. *Probe*: Has it changed over time? If so, what do you believe has contributed to this change? 4. What do you find most rewarding in your role? What do you find most challenging? 5. What would your ideal job description be? 6. From your perspective, what are some of the biggest challenges or issues impacting EMS’s ability to deliver high-quality and efficient care? 7. Some of the policy directions identified by Alberta Health include adding more ambulances and hiring more staff. Do these address these challenge areas? Why or why not?	Are you registered as a paramedic in Alberta? How long have you been working with EMS? What is your current practice setting? Urban, rural or both? Note: Rural is defined as a community with a population under 25,000. Would you like your job description to change? If so, what would be the feasible policy changes to enable this? What is your vision for the profession in 10 years? What keeps you in this profession? Response times, Staffing and Emergency Department offloading have been identified as key concerns. Are there other areas that you see as important?
Response Times	1. Response times have been reported to be increasing in length in recent years. Have you noticed this? If so, when did you begin to notice the increasing response times? 2. What impact do long response times have on patient care? 3. How has the provincial “borderless” system impacted the ability of paramedics to respond to medical emergencies? 4. What impact do lower acuity calls have on your ability to provide care to higher acuity calls?	What factors do you believe are contributing to longer response times? What is the impact of red alerts when they occur? What could be done to reduce long response times? How might multiple provincial health authorities impact this? Note: a provincial health authority is a publicly funded health service provider (i.e. AHS, or Calgary Health Region) What does this look like in a rural setting? What are your thoughts on using Poison and Drug Information System (PADIS) or 811 to redirect low-acuity calls? Has the recent change to not automatically dispatch an ambulance to motor vehicle collisions (MVCs) effectively reduced unnecessary calls? Why or why not? Have you noticed whether the utilization of taxi or private services impacted call volume? If so, how? What possible solutions do you have to reduce or address low-acuity calls?
Staffing	1. Please tell me about the staffing model in EMS. 2. What are the barriers or challenges impacting staffing in EMS? 3. Do you believe the culture of EMS has impacted staffing? If so, how? 4. How has the hiring of new paramedics impacted the workload? 5. It has been noted that recruiting and retaining paramedics in rural locations has been a challenge. What makes staffing in rural areas challenging? 6. How do EMS services work on reserves and settlements in Alberta?	What changes, if any, have you noticed in recent years to the staffing model? It has been reported that nearly 19,000 paramedic shifts went unfilled in Alberta in 2022. What are some strategies to reduce shift vacancies? Do you believe staffing models impact red alerts? If so, how? Can you list the contributing factors impacting culture? How would you rank these contributors from most impactful to least? What impact do these new hires have on existing practitioners? What would make you want to work in a rural setting? What are some possible solutions to addressing short staffing in rural areas? What is the EMS coverage on reserves and settlements like? What are some challenges of EMS delivery on reserves and settlements? Could EMS delivery be improved on reserves and settlements? If so, how could EMS better support reserves and settlements in Alberta?
Emergency Department Offloading	1. How do Emergency Department wait times impact EMS resources? 2. What is the relationship between primary care and EMS?	What do you believe is contributing to this? Do you feel transferring patients to low-acuity Emergency Department teams will facilitate a faster return to service? Do you believe paramedics waiting in the hallways is a concern? If so, what are your thoughts on reducing the time paramedics spend waiting in the hallways with patients? Did you ever notice when Emergency Department wait times impacted offloading? The Canadian Medical Association reported a critical family medicine shortage. How do challenges with primary care access impact call volumes? What are some ideas for collaboration between EMS and primary care? Are there alternative clinics or resources that could redirect patients from the Emergency Department? If so, how could this be facilitated? What does this look like in a rural setting?
Conclusion	1. Are any other comments you have that were not brought up in this interview that you believe need to be addressed?	

### Data analysis

Interviews were transcribed verbatim using Rev software. Interpretive description methods guided data analysis, with a focus on seeking to answer experiential questions with relevant output for practical use. Thematic analysis was used to analyze the interview data where inductive coding of interview transcripts was done using NVivo (version 12.0) [14]. Data was coded into themes and subthemes in a codebook (labelled codes and definitions) by the corresponding researcher. Codes were categorized and coalesced themes and subthemes inductively with the research team through discussion and review to ensure confirmability [15]. Rigor was supported by evaluating and validating codes, themes and subthemes with the research team. To establish trust in the findings, peer debriefing was used to validate codes and themes with the project team members [15].

### Trustworthiness

Purposeful sampling was utilized to select a maximum variation of paramedics from across Alberta with varying levels of practice experience. The credibility of the research findings was reviewed by a research associate and two expert supervisors. This was done to ensure valid themes and conclusions from the data obtained.

### Ethical considerations

Participants were informed of the study’s purpose and the benefits of participating in research before each interview. Participation was voluntary and no compensation was provided for participation. Before the interview, participants were made aware that they could withdraw their participation at any time during the interview and up to 7 days after the interview without negative consequences. Privacy and confidentiality was upheld through hypothetical codes and the removal of identifying information from interview recordings.

## Results

### Characteristics of sample

A total of 19 regulated primary care or advanced care paramedics were interviewed, with sociodemographics described in Table 2. Interviews were held virtually for an average of 62 min between April and May 2023. Participants were diverse, with experience in both rural and urban areas, varying career lengths, and experience in a multitude of different roles (from volunteer positions to combination services and managerial roles), representing a holistic frontline provider perspective.

**Table 2:** Participant demographics in 19 participants

Variables	Absolute (#)	Percentage of total (%)
Gender (Man/Woman)	12/7	63
Designation (Advanced Care Paramedic/Primary Care Paramedic)	12/7	63
Most Recent Practice Setting (Urban/Rural/Both)	11/5/3	58/26/16
Years in EMS		
0–5 yrs 6–10 yrs 11–15 yrs 16–20 yrs 21–25 yrs 26–30 yrs 31–35 yrs 35–40 yrs	4 3 3 4 1 3 0 1	21 16 16 21 5 16 0 5

### Findings from qualitative interviews

**Fig. 1:**
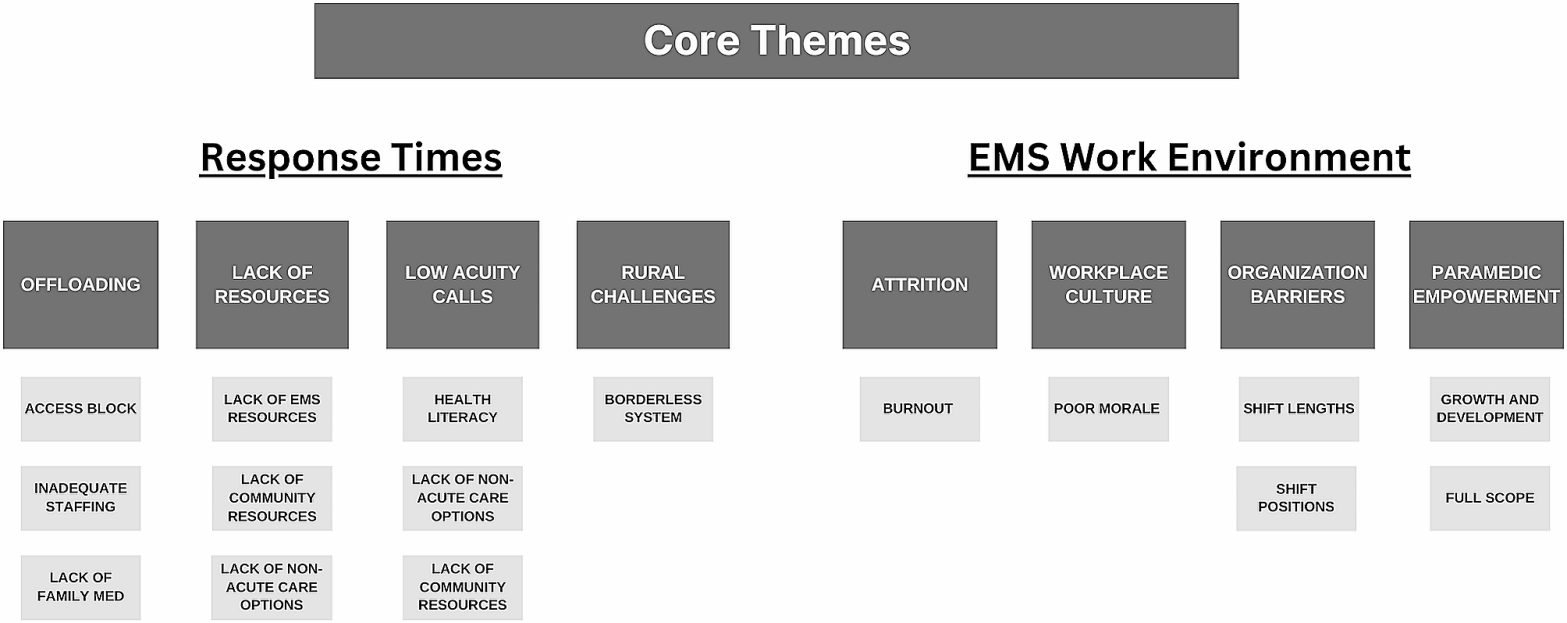
Paramedic identified system concerns

**Fig. 2:**
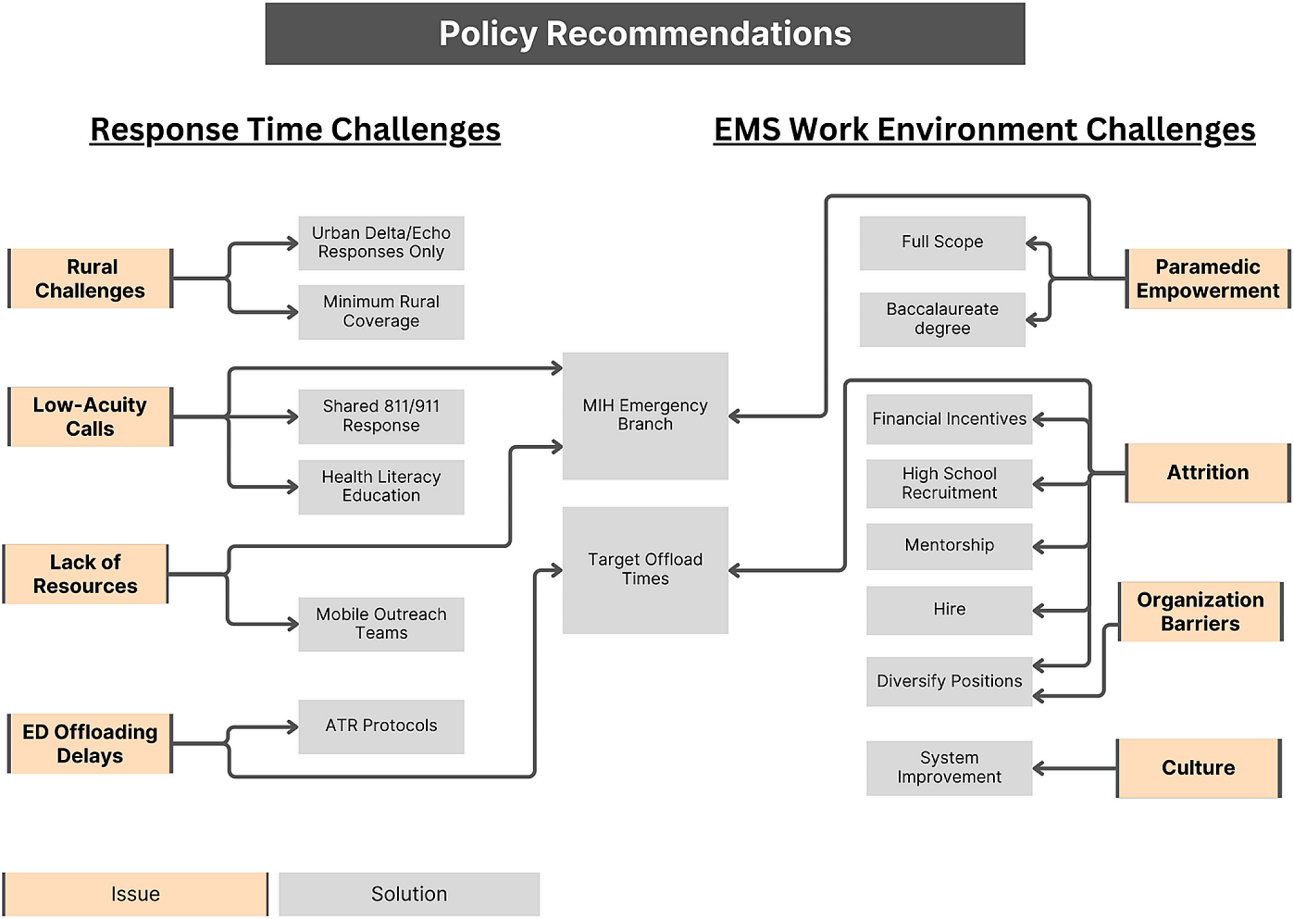
Policy recommendations based on core themes

Findings indicated that participants felt that the EMS system in Alberta was at its breaking point, with serious concerns about its longevity. Overall, call volumes were perceived to overwhelm EMS resources. More specific findings focused on core themes: (1) contributors to poor response times and (2) a deteriorating EMS working environment (Fig. 1), which led to some policy recommendations to address the issues identified (Fig. 2; Tables 3 and 4).

**Table 3:** Emblematic quotes and provider-suggested solutions for response times

Solutions	Description	Quote(s)
Implement and enforce provincial hospital offload target times Expand ATR protocols	Offload targets incentivize against inappropriate and counterproductive utilization of EMS staff at hospitals for large proportions of shifts. A recently implemented 45-minute target in Alberta has been considered an effective strategy by a majority of practitioners. Practitioners need to be able to provide treatment on scene and refer patients for alternative follow-up, without hospital assessment where possible. A new January 2023 general assess, treat, and refer (ATR) protocol for practitioners was perceived as successful in preventing unnecessary transport to EDs or Urgent Care Centres (UCCs). Where high-risk factors are present, paramedics need to be able to consult a physician via On-Line Medical Consultation (OLMC) or similar means.	“[There needs to be] *ways to figure out how to resolve bed block within hospitals. People waiting for long-term care back up the borders of emergency, which backs up the ability to get people, which backs up throughput through the emergency department”(P18).* *“We’re seeing that now with that general ATR protocol where we can actually tell people to stay at home… I’ve used that quite a few times. Whereas the default… still is to transport, but we’re not supposed to tell a 100% of our patients to go to the hospital” (P4).* “*Trying to decide whether* [the patient should*] go to the hospital. Now I’m calling OLMC to get advice whether* [the patient] *should go in, or stay.” (P9).*
Avoid deprioritizing EMS relative to emergency departments Ensure adequate staffing numbers Increase provision of additional community resources	Regulations and practices need to change to proactively prevent use of EMS providers as a captive workforce to supplement overburdened EDs. Paramedics need to be able to serve in their intended roles unless specifically assigned to ED-specific roles. Need to improve recruitment and retention strategies, and decrease attrition. There is significant need for addiction treatment or mobile outreach teams that respond to street-level intoxication and unhoused individuals to allow EMS to focus on core health roles.	*“When an ambulance brings a person to the ED and they don’t get to offload and get back into service right away, that impacts availability of the resource. Then the other big question, “Is it the best place for the patient, best outcome?” (P11)* *“Downtown clientele… have larger social programming needs. They have addiction problems. They have trauma and they don’t need the hospital” (P19)*
Target health literacy education Share 811/911 response Create an emergency mobile integrated health (MIH) branch	Inappropriate use of emergency services needs to be reduced. Public education and self-direction to more appropriate services may have some benefit. Low acuity calls often require more time and resources than higher ones. More effective 811 may reduce emergency calls. This needs to include prioritization of reducing ‘bounce back’ of low acuity 811 calls, especially by effective integration of physicians into the 811 system and increasing available response options. While capable of providing some limited immediate assistance to patients, EMS services lack the resources or scope of practice to help more effectively or definitively. Mechanisms to facilitate mobile definitive care are needed.	*“[The general public needs education] on when to call an ambulance and when not to” (P5).* *“[When providers are dealing with low acuity calls for prolonged periods, we] could be providing a higher level of care to someone in cardiac arrest two blocks away” (P5)*
Rural Challenges	The is a need to impose guardrails to ensure rural ambulances are not used consistently to support urban areas. Participants referenced First Nations (with independent EMS services), responding outside their geographic area only if the call is a Delta or Echo (the highest acuity calls). Need to evaluate current ambulance optimization algorithms to account for Alberta context including geographic and staffing shortages.	*“My ambulance that belongs to my town and my area is very often gone. It’s gone. It’s in Calgary, it gets sucked into the vortex, and it never comes back, but we can’t escape” (P15).* *“[There] should be a bare minimum [of ambulances to ensure minimum coverage of rural communities” (P10).*

**Table 4:** Emblematic quotes and provider-suggested solutions for EMS working environment

Solutions	Description	Quote(s)
Reduce excessive workloads Increase hiring but prioritize retention Incorporate more off-service time for education Increased financial incentives for rural practitioners Better targeted recruitment of the next generation of paramedics	The status quo of managing immediate challenges only is counterproductive, longer term solutions are needed. Increased hiring was stressed to be unlikely on its own to result in quick improvements to working conditions. New hires would benefit from the implementation of structured mentorship programs (i.e. Formal roles where staff could exercise leadership and directly provide better support to providers) to help with retention and address perceived gaps in knowledge. Current incentives may not be adequate. Increased incentives may provide benefit from compensating any expanded roles necessary in rural areas. More active and earlier recruitment is needed including targeting high school students, particularly in rural communities. Participants advocated specifically that grant funding be allocated to rural communities to train high school students as medical first responders, which would provide a starting block to a career in EMS. This was also mentioned as a potentially useful strategy for enhancing care in Indigenous communities, especially coupled with increased supports and scope of practice changes to facilitate community-based care.	*“We’re having a huge influx of very new practitioners and a huge exodus of the old, experienced practitioners” (P1).* *“Consequentially, we lose a lot of our new staff because the environment that they’re coming into is… it’s so toxic, and it’s so broken”(P15).* *“I think better benefits and things like health benefits and spending accounts and things. I mean, those always attract people. Obviously more money. If you pay them more than the cities, then chances you’re able to attract at least some people out there” (P4).* *“Bring in some of these local Indigenous students, who would be just incredibly phenomenal in a paramedic role and especially in some kind of community paramedicine role really targeted into their communities and their homes.” (P11).*
Workplace Culture Improvements Require Broader Progress First	Paramedics expressed near universal burnout within the profession. Significant psychologic effects of an EMS system perceived to be crumbling, associated moral injury and feelings of powerlessness were described. Providers indicated direct progress on changing culture in the near-term is likely to be infeasible. They could not propose any interventions on culture directly that were thought to have any potential efficacy.	*“I think we work our full-time employees really hard, and they either burn out, which results in absenteeism, or they burn out, and they just become kind of emotionally shut down at work” (P16).*
Change shifting patterns Eliminate the Core-Flex staffing model in rural settings (often scheduling paramedics for 96 h at a time)	Providers need more flexibility including part-time options and options for variable shift lengths. Consider establishing more flexible roles within the ED in rural areas, which would have potential advantages of facilitating learning and development, improving job satisfaction and helping address broader profound staff shortages in rural Eds.	*“People don’t want to work full-time. So, trucks can’t reliably be staffed, which is the issue I’ve seen the most. And it just leads to an already under-resourced system losing more resources, which feeds into a negative feedback loop”(P18).* *“Getting rid of core-flex but still having available housing to stay there. Core-flex is a nightmare. Nobody should have to work it, ever. I think that going to a fire-style schedule that has 24 hours on, 24 hours off, 24 hours on and five days off, would lend to more staff being inclined to go and stay for a 24-hour shift and then have almost a week off every time”(P10).*
Expand competencies and roles for the profession Define and expand “*blended emergency response and clinical-based” (P7)* roles	Participants suggested converting the two-year ACP Diploma to a four-year baccalaureate degree – bringing further credibility and rigour. More training also expands possibilities for use of new competencies and capabilities. Creating mentorship and leadership roles will allow paramedics greater direct capability to combat attrition and manage the destiny of their environment. The relative skills rural paramedics have are often underutilized – expanded clinical roles and responsibilities could bolster human resources in small communities, improve access to care and potentially reduce the transfers of patients to more specialized facilities. Expansion of MIH and ATR protocols would expand appropriate utilization of paramedic skills (concurrently hopefully reducing morale-draining underutilization) and reduce ED visits.	*“Having our PCPs and our ACPs working at their full scope, moving to two-year diplomas and four-year degrees as the default for those qualifications” (P7).* *“There’s a huge, huge potential for us to, not only through community paramedicine, the MIH initiatives, but with our treat and refer programs, for us to leverage our scope more fully with telemetry, with virtual communications.” (P7)*

### Response times

Response times are described as the time between when a 911 call is made and the time an ambulance arrives. One participant usefully summarized how response times can be broken down into three categories: event identification before dispatch, “chute time” from when dispatched, and then the travel time to the event. In the overall context of research findings, poor response times were described as an emblematic indicator of whether paramedics were able to effectively fulfill their intended roles and responsibilities. There was virtually unanimous agreement that response times in Alberta are too long, however, there were different perspectives on the causes. Participants frequently shared that response times can be delayed when the dispatcher doesn’t have a close available unit because crews are still in the ED providing care to their patient(s). Some viewed this as a long-standing issue while others thought response times were much worse just before the pandemic. Overall, participants identified four contributing factors to long response times: ED offloading delays, lack of resources, low-acuity calls, and rural challenges (Fig. 1). Some emblematic quotes and descriptions of identified policy recommendations are described in Table 3.

With respect to offloading in the ED (the transfer of care), paramedics confirmed widespread difficulty handing over care to ED staff, preventing them from being available to respond to further emergencies. Participants cited three main factors preventing timely offload including (a) access blocks, (b) inadequate ED staffing, and (c) lack of non-acute options to provide alternative venues of care for appropriate patients. Participants reported that the broader lack of available ambulances coupled with this disfunctional detention of ambulances in EDs compounded moral injury and guilt about not being able to provide appropriate care to high acuity patients. Furthermore, focused dismay was expressed at the inaccessibility of primary care, addiction services and housing programs creating strain on EMS who, while capable of providing some assistance to patients immediately in front of them, lack the resources or scope of practice to help more effectively or definitively. Low acuity calls were perceived as requiring the same or much longer time commitments than higher acuity ones, compounding the adverse effects of this mismatch. Participants described in detail the EMS working environment including the physical, social, and emotional space and conditions experienced by paramedics and the elements that effect their ability to perform their core responsibilities.

Interestingly, when participants were probed regarding challenges in rural communities, known difficulties with rural recruitment and retention were not their main concern. The previously described borderless system (despite its perceived potential benefits) was viewed as a predominant player in exacerbating the crisis in rural areas. Participants confirmed impressions that striping vulnerable areas of their (limited) available units and subsequent ongoing detention in the cities was uniquely counterproductive – with significant undesirable knock-on effects for existing crises in recruitment and retention.

### Working environment

In reponse to prompts touching on the working environment and how this impacts operations, participants focused on four aspects of the EMS working environment: attrition, workplace culture, organization barriers and paramedic empowerment.

Paramedics expressed near universal burnout within the profession. This resulted from the overburdensome demands of current operations but also the psychological effects of an EMS system perceived to be crumbling and the moral injury and feelings of powerlessness. Attrition was viewed as a direct consequence, especially for new providers. The exodus of new providers itself was thought to compound this problem further. These dismal realities were thought to contribute to a toxic culture overall, namely one where provider well-being is given no value, expectations are to address short-term considerations above all else, and a mentality of survival is perceived as emphasized over clinical excellence. These attitudes, beliefs, and norms were felt to comprise the workplace culture. Providers indicated the situation is likely so fargone that direct progress on changing culture in the near-term is likely to be infeasible without notable progress in other areas. Strikingly, participants could not propose any interventions on culture.

Thirteen participants spoke about rigid organizational structures impairing staffing. Specifically, there is limited variability in shift lengths and positions. Most felt that current imposed standards of full-time work, 12 h a day, four days on and four days off could not lead to a schedule that enabled them to live a balanced life or care adequately for their families. Flexibility in hours and roles was thought to contribute directly to potentially improving staffing levels, but also indirectly by serving to facilitate an expansion of EMS scope of practice with further beneficial effects (Table 4). Strikingly, desire for role redefinition and empowerment was widely held, while preference for specific items varied amongst participants. Efforts at facilitating empowerment and professional development, especially structuring mentorship and peer supports, were viewed as a particularly critical first step and raise hopes of a healthier culture being possible.

## Discussion

This research study confirms that response times are emblematic of the primary role paramedics are expected to fill. Paramedics expressed significant distress with lengthening response times symbolizing EMS system failures, direct adverse effects on patient care and the overall healthcare system that is over capacity. As discussed, worsening response times reflect many interconnected factors and are unlikely to improve markedly without coordinated action. The issue of ED overcrowding is one symptom paramedics deal with on a daily basis, but reflects the overall system failure from government underfunding and the simultaneous erosion of primary care [16, 17]. For example, current policies aimed to specifically reduce offload delays will fail to produce sustained improvements if ED throughput is not improved as ED overcrowding is a root cause of offload delays [6]. With the rapid growth of Albertans and the aging of the population, healthcare needs have outpaced current resources [18]. The lack of hospital beds in Alberta can be traced back to 1993–1995 when the amount of hospital beds was approximately halved by the Alberta government [19]. Since this policy decision the amount of acute care beds per 100, 000 has continued to decline and never returned to previous acute care beds in the 1980s [19, 20]. From the paramedic perspective, interventions like better primary care access could result in more meaningful benefits if call volumes, hospital transports or ED visits are reduced as a result [21]. Evidence supports further recommendations to expand services connecting patients to more appropriate settings and services, reducing costs and likely improving care [22]. A recent and promising Ontario study demonstrated that utilizing MIH (mobile integrated health) to meet the urgent care needs of communities can be a successful care and cost-effective delivery method [23].

In the broader context, our study highlights that paramedics desire and need more transformational change. Unfortunately, current policy interventions are too often incremental and hyperfocused on modest near-term improvements. Exemplifying this, the Alberta government had previously assembled the Alberta EMS Provincial Advisory Committee who delivered a final report in fall 2022. This report sought provider input resulting in rich and detailed feedback mirroring the interview responses obtained in our study [24]. Unfortunately, the final 10-point list of approved recommendations focuses predominantly on modest adjustments like decreasing mandatory EMS attendance at fires, forming a task force to further investigate ED-related issues, constructing new and more clear transfer of care guidelines for EMS offloads, and improving the health literacy of Albertans. Despite discussing more complex problems at length, the final recommendations lack more specific transformational measures to enhance staffing, scheduling, recruitment, professional development and burnout.

While much more difficult, truly addressing this crisis in EMS requires more fundamental changes in how EMS and the broader health system operates, specifically empowering and improving the well-being of paramedics. This is not limited to Alberta alone, but within Canada and other jurisdictions internationally have similar concerns [25, 26]. Addressing cultural and operational issues like burnout and sky high attrition requires understanding how policies can contribute to a more positive and effective workplace culture in the medium term and a more capable and reliable labour force in the long term. Investments in training, wellness and mentorship pathways may be difficult to implement in short-term resource-constrained environments, but the literature does support the importance of mentorship, professional development, and retention [27, 28]. Such approaches require nuance and local adaptation, necessitating identifying and empowering local champions to pursue strategies like preferential recruitment and support for rural students that evidence shows are more likely to be retained as rural healthcare workers [29].

Furthermore, the solutions we have highlighted as desired by paramedics attest to the need to change the acute care focus of Canadians and the Canadian acute care system. Changing scope of practice for paramedics, enhancing outpatient services and providing better access to alternative venues of care may reduce the overdependence on EDs and acute care hospitals [30]. In an environment where primary care providers especially are in short supply, a hopefully re-invigorated and expanded paramedic workforce could offer an effective alternative pool of talent and skills to contribute to this transition. Evidence from the UK recently looked into the expansion of the paramedic role into primary care from patient, physician and paramedic perspectives. The outcome of that systematic scoping review highlighted how paramedics could spend additional time with patients, however, there were mixed perspectives [31]. While the reality of the situation is that EMS, along with other healthcare practitioners, are facing demands that far exceed the capacity of the healthcare system itself which is contributing to downstream areas such as EMS slowing response times and creating a poor working environment.

### Strengths and limitations

Limitations of this research project include non-random sampling, limited timeline, and the virtual nature of the interview. One of the limitations was that participants were recruited via social media, limiting the pool of possible practitioners who could be reached. Furthermore, this study only interviewed frontline paramedics which represents one perspective within a complicated health system of providers and patients. In addition, due to project time constraints, interviews all occurred over a month and were limited based on response rates. Another limitation is that all interviews were conducted virtually, which sometimes caused delays or unintentional interruptions, which can impact the interview ambience. However, the notable strengths of this study includes adding evidence to a relatively underresearched area and using a solution based approach to interview design.

## Conclusion

Despite the participants’ concerns about poor response times, an unhealthy EMS working environment and the crushing challenges facing the profession overall, practitioners also spoke about the hope they had for the profession. They frequently spoke about the unique environment they work in and their love of caring for their patients. We should support them and their colleagues with a continued and sincere willingness to reform and enhance the pre-hospital system and enable the profession to grow and improve. Further research investigating MIH initiatives in reducing hospital transfers as well as the availability of units and clinical outcomes when offload times are mandated and enforced is recommended.

### Electronic supplementary material

Below is the link to the electronic supplementary material.

Supplementary Material 1

Supplementary Material 2

## Data Availability

The corresponding author (JN) can be contacted about the data with restriction. All data generated or analyzed during this study are included in this published article. Raw interview data cannot be shared due to expectations regarding privacy and the research ethics board.

## Abbreviations

ACP: Advanced Care Paramedic
ED: Emergency Department
EMS: Emergency Medical Services
MIH: Mobile Integrated Health
PCP: Primary Care Paramedic
