# Supplementary material for: Exploring paramedic perspectives on emergency medical service (EMS) delivery in Alberta: a qualitative study
Source: BMC Emerg Med. 2024 Apr 16;24:66. doi: 10.1186/s12873-024-00986-z (PMC11020468; doi:10.1186/s12873-024-00986-z)
Supplement: Supplementary file 1 — Supplementary Material 1 [file 12873_2024_986_MOESM1_ESM.docx]

**Appendix A**

Table 1. Interview Theme Description

| **Themes** | **Definition** |
| --- | --- |
| Response Times | Seeks to elucidate the prevalence of long response times and to explore how policies have impacted the length of response times. Explore how alternative care models may impact and reduce low-acuity EMS calls. |
| ED Offloading | Focus on how continuing care of patients in the hospital impacts EMS resource availability. Seek paramedic perspectives on how safe transfer of care for low acuity patients can be facilitated. |
| EMS Working Environment | Understanding how staffing is structured, culture and new hires have impacted the workplace. Explore solutions to fill shift vacancies in urban and rural settings. |
