# Supplementary material for: Exploring paramedic perspectives on emergency medical service (EMS) delivery in Alberta: a qualitative study
Source: BMC Emerg Med. 2024 Apr 16;24:66. doi: 10.1186/s12873-024-00986-z (PMC11020468; doi:10.1186/s12873-024-00986-z)
Supplement: Supplementary file 2 — Supplementary Material 2 [file 12873_2024_986_MOESM2_ESM.docx]

**Appendix B**

**Interview Script**

***Warm-up***

1. Can you tell me what your designation is? OR You indicated that you are a PCP/ACP, can you confirm that this is correct?

*Probe:* Are you registered as a paramedic in Alberta?

1. Can you please tell me about your role(s) with EMS?

*Probe:* How long have you been working with EMS?

*Probe:* What is your current practice setting? Urban, rural or both? *Note:* Rural is defined as a community with a population under 25,000.

1. In your time working for EMS, what has the culture been like? *Note:* Culture here is defined as a collection of attitudes, beliefs, and behaviours that make up the workplace environment.

*Probe:* Has it changed over time? If so, what do you believe has contributed to this change?

1. What do you find most rewarding in your role? What do you find most challenging?
2. What would your ideal job description be?

*Probe:* Would you like your job description to change? If so, what would be the feasible policy changes to enable this?

*Probe:* What is your vision for the profession in 10 years?

*Probe:* What keeps you in this profession?

1. From your perspective, what are some of the biggest challenges or issues impacting EMS’s ability to deliver high-quality and efficient care?

*Probe:* Response times, Staffing and Emergency Department offloading have been identified as key concerns. Are there other areas that you see as important?

1. Some of the policy directions identified by Alberta Health include adding more ambulances and hiring more staff. Do these address these challenge areas? Why or why not?

***Response Times***

1. Response times have been reported to be increasing in length in recent years. Have you noticed this? If so, when did you begin to notice the increasing response times?

*Probe:* What factors do you believe are contributing to longer response times?

1. What impact do long response times have on patient care?

*Probe:* What is the impact of red alerts when they occur?

*Probe:* What could be done to reduce long response times?

1. How has the provincial “borderless” system impacted the ability of paramedics to respond to medical emergencies?

*Probe:* How might multiple provincial health authorities impact this? *Note:* a provincial health authority is a publicly funded health service provider (ie. AHS, or Calgary Health Region)

1. What impact do lower acuity calls have on your ability to provide care to higher acuity calls?

*Probe:* What does this look like in a rural setting?

*Probe:* What are your thoughts on using Poison and Drug Information System (PADIS) or 811 to redirect low-acuity calls?

*Probe:* Has the recent change to not automatically dispatch an ambulance to motor vehicle collisions (MVCs) effectively reduced unnecessary calls? Why or why not?

*Probe:* Have you noticed whether the utilization of taxi or private services impacted call volume? If so, how?

*Probe:* What possible solutions do you have to reduce or address low-acuity calls?

***Staffing***

1. Please tell me about the staffing model in EMS.

*Probe:* What changes, if any, have you noticed in recent years to the staffing model?

1. What are the barriers or challenges impacting staffing in EMS?

*Probe:* It has been reported that nearly 19,000 paramedic shifts went unfilled in Alberta in 2022. What are some strategies to reduce shift vacancies?

*Probe:* Do you believe staffing models impact red alerts? If so, how?

1. Do you believe the culture of EMS has impacted staffing? If so, how?

*Probe:* Can you list the contributing factors impacting culture?

*Probe:* How would you rank these contributors from most impactful to least?

1. How has the hiring of new paramedics impacted the workload?

*Probe:* What impact do these new hires have on existing practitioners?

1. It has been noted that recruiting and retaining paramedics in rural locations has been a challenge. What makes staffing in rural areas challenging?

*Probe:* What would make you want to work in a rural setting?

*Probe:* What are some possible solutions to addressing short staffing in rural areas?

1. How do EMS services work on reserves and settlements in Alberta?

*Probe:* What is the EMS coverage on reserves and settlements like?

*Probe:* What are some challenges of EMS delivery on reserves and settlements?

*Probe:* Could EMS delivery be improved on reserves and settlements? If so, how could EMS better support reserves and settlements in Alberta?

***Emergency Department Offloading***

1. How do Emergency Department wait times impact EMS resources?

*Probe:* What do you believe is contributing to this?

*Probe:* Do you feel transferring patients to low-acuity Emergency Department teams will facilitate a faster return to service?

*Probe:* Do you believe paramedics waiting in the hallways is a concern? If so, what are your thoughts on reducing the time paramedics spend waiting in the hallways with patients?

*Probe:* Did you ever notice when Emergency Department wait times impacted offloading?

1. What is the relationship between primary care and EMS?

*Probe:* The Canadian Medical Association reported a critical family medicine shortage. How do challenges with primary care access impact call volumes?

*Probe:* What are some ideas for collaboration between EMS and primary care?

*Probe:* Are there alternative clinics or resources that could redirect patients from the Emergency Department? If so, how could this be facilitated?

*Probe:* What does this look like in a rural setting?

***Conclusion***

1. Are any other comments you have that were not brought up in this interview that you believe need to be addressed?
